# Deploying machine learning models in clinical settings: a real-world feasibility analysis for a model identifying adult-onset type 1 diabetes initially classified as type 2

**DOI:** 10.1093/jamiaopen/ooaf133

**Published:** 2025-10-26

**Authors:** Irene Brusini, Suyin Lee, Jacob Hollingsworth, Amanda Sees, Matthew Hackenberg, Harm Scherpbier, Raquel López-Díez, Nadejda Leavitt

**Affiliations:** AI for Healthcare & MedTech, IQVIA, London W2 1AF, United Kingdom; AI for Healthcare & MedTech, IQVIA, Wayne, PA 19087, United States; AI for Healthcare & MedTech, IQVIA, Wayne, PA 19087, United States; AI for Healthcare & MedTech, IQVIA, Wayne, PA 19087, United States; AI for Healthcare & MedTech, IQVIA, Wayne, PA 19087, United States; HealthShare Exchange (HSX), Philadelphia, PA 19106, United States; Breakthrough T1D (Formerly JDRF International), New York, NY 10281, United States; AI for Healthcare & MedTech, IQVIA, Wayne, PA 19087, United States

**Keywords:** type 1 diabetes mellitus, machine learning, clinical decision support systems, electronic health records, Health Information Exchange

## Abstract

**Objective:**

This study evaluates the performance and deployment feasibility of a machine learning (ML) model to identify adult-onset type 1 diabetes (T1D) initially coded as type 2 on electronic medical records (EMRs) from a health information exchange (HIE). To our knowledge, this is the first evaluation of such a model on real-world HIE data.

**Materials and Methods:**

An existing ML model, trained on national US EMR data, was tested on a regional HIE dataset, after several adjustments for compatibility. A localized model retrained on the regional dataset was compared to the national model. Discrepancies between the 2 datasets’ features and cohorts were also investigated.

**Results:**

The national model performed well on HIE data (AUROC = 0.751; precision at 5% recall [PR5] = 25.5%), and localization further improved performance (AUROC = 0.774; PR5 = 35.4%). Differences in the 2 models’ top predictors reflected the discrepancies between the datasets and gaps in HIE data capture.

**Discussion:**

The adjustments needed for testing on HIE data highlight the importance of aligning algorithm design with deployment needs. Moreover, localization increased precision, making it more appealing for patient screening, but added complexity and may impact scalability. Additionally, while HIEs offer opportunities for large-scale deployment, data inconsistencies across member organizations could undermine accuracy and providers’ trust in ML-based tools.

**Conclusion:**

Our findings offer valuable insights into the feasibility of at-scale deployment of ML models for high-risk patient identification. Although this work focuses on detecting potentially misclassified T1D, our learnings can also inform other applications.

## Background and significance

Type 1 diabetes (T1D) and type 2 diabetes (T2D) differ in clinical presentation and progression.^1^ However, initial misclassification is common, with over 40% of adults developing T1D after age 30 years initially treated as having T2D.^2–4^ This is firstly due to the traditional paradigm associating T1D with juvenile onset, and T2D with adult onset, despite both conditions occurring in both age groups.^1^^,^^5–7^ Additionally, the initial presentation of adult-onset T1D can resemble that of T2D,^2^^,^^5^^,^^8^ and common clinical indicators (eg, body mass index [BMI] and metabolic syndrome) can be poor discriminators.^5^^,^^6^^,^^8^ Adult diabetic patients are often under the management of a primary care provider, and lack of specialty interactions could also increase misclassification risk.^9^ Misclassifying T1D as T2D can lead to delayed or ineffective treatment, causing prolonged hyperglycemia and increased risk for adverse outcomes like diabetic ketoacidosis.^1^^,^^2^ Therefore, it is important to investigate novel approaches to assist clinicians in accurate early diagnosis of T1D in adults.

The increasing digitalization in healthcare and the availability of large electronic medical record (EMR) datasets have enabled the development of machine learning (ML) models for disease detection,^10–13^ aiming to identify high-risk patients for early intervention. ML-based solutions have been proposed to better identify and manage T1D. Daniel et al^14^ implemented an ensemble model to identify T1D in children using primary care EMRs. Andersen et al^15^ proposed a logistic regression (LR) model to predict T1D-related comorbidities and mortality in adults using registry data. LR was used also by Lynam et al^16^ to distinguish T1D from T2D in adults, therefore addressing the challenge of classifying diabetes type. However, this model was only tested on limited cross-sectional datasets, comprising White European patients aged 18-50. A broader and more heterogenous patient population was used by Cheheltani et al^9^: a US national EMR dataset, including more than 4 million diabetic patients of diverse ethnicity and age, was leveraged to train an XGBoost classifier^17^ to predict adult-onset T1D initially coded as T2D. Differently from LR, XGBoost is powerful at handling missing data, a property that can widen its applicability on real-world EMR datasets.^18^

While these ML-based solutions show promise for integration into clinical decision support tools (CDSTs),^19^ their clinical uptake is still slow and limited.^20^ A major challenge to real-world deployment lies in the discrepancies between the development and deployment datasets,^18^^,^^21^ which can reduce predictive accuracy, undermining providers’ trust and adoption of these tools. Therefore, to assess deployment feasibility, ML algorithms must be validated on external datasets that reflect the demographics and care patterns of target deployment data.^22^ While some ML models for T1D identification have been tested on external research datasets,^14^^,^^16^ validation is still lacking—to the best of our knowledge—on real-world datasets from healthcare organizations (HCOs) where the models are eventually going to be deployed.

This study aims to close the existing gaps in the literature and to establish a path towards scaled deployment of ML CDSTs to aid clinicians in accurately diagnosing adult-onset T1D. This was achieved by testing the model proposed by Cheheltani et al^9^ on an external dataset provided by a health information exchange (HIE) organization. HIEs allow multiple HCOs to securely share and access patients’ EMRs, improving the completeness of patient records and, therefore, their quality of care.^23^ As such, they also hold great potential for deployment of ML algorithms at scale across multiple HCOs within the same HIE network. To the best of our knowledge, this is the first study to test an ML-based solution for identifying adult-onset T1D initially coded as T2D using real-world HIE data for deployment purposes.

## Objectives

Three main objectives were addressed. First, the original algorithmic design^9^ was revisited to address both the requirements for clinical deployment and the discrepancies between the national training dataset and the regional HIE deployment dataset. Second, the revisited ML model was tested on HIE data to analyze its generalizability and transportability. Lastly, a new “localized” model was retrained on the HIE dataset, to examine whether retraining on a smaller local dataset—which more closely reflects deployment data—is beneficial over leveraging large-scale EMR training datasets. By addressing these goals, this study also sheds light on the benefits and challenges of leveraging large-scale EMRs for clinical ML model development, particularly when the target deployment data comes from an HIE representing a regional portion of the patient population. Moreover, it explores the potential of deploying ML models on HIEs for high-risk patient identification, using lessons drawn from the present use case of detecting potentially misclassified adult-onset T1D.

## Materials and methods

### Datasets

The national IQVIA Ambulatory EMR (AEMR) database comprises records from approximately 97 million patients who had physician interactions in a network of over 100 000 physicians affiliated with over 800 ambulatory practices from all US states. AEMR data captured between 1 July 2014 and 30 April 2023 were used to train the revisited national ML model.

A regional dataset provided by HealthShare Exchange (HSX) was then used to both validate the national model and train the new localized model. HSX is an HIE that links EMR data from a network of hospitals and health systems in the Greater Philadelphia region. HSX records between 1 January 2016 and 31 December 2022 were used for this study.

Both the AEMR and the HSX datasets capture diagnoses, prescribed medications, clinical procedures, laboratory (lab) test results, vital signs, encounters and demographics, recorded using a combination of structured codes and free text inputted by physicians. However, while the AEMR dataset includes complete EMRs from all practices, data coverage across different types of records received by HSX can vary across HCOs. Therefore, certain types of clinical events may be missing at different rates for some patients.

### Overview of the original algorithm

In the study by Cheheltani et al,^9^ cohorts were built from the AEMR by identifying the date of first T2D diagnosis (*index date*) for all patients aged 18 or over with no prior T1D record. Features were then engineered from a set of selected clinical events in the 2 years prior to the index date (the *lookback period*). Patients were labeled as either *positive* or *negative* depending on whether they did or did not receive, respectively, a T1D diagnosis (not followed by any T2D diagnosis) within 3 years after the index date (*outcome period*). Finally, an XGBoost classifier was trained to identify T1D patients that were misclassified as T2D at the index date.

In the present work, some amends were made to the original methodology to successfully carry out an external validation and improve the model’s fitness-for-use on the HIE dataset. The most substantial changes are discussed in the next section, and more details can be found in the Supplementary Material.

### Algorithmic improvements for HIE deployment

#### Patient cohorts

**Figure 1.**
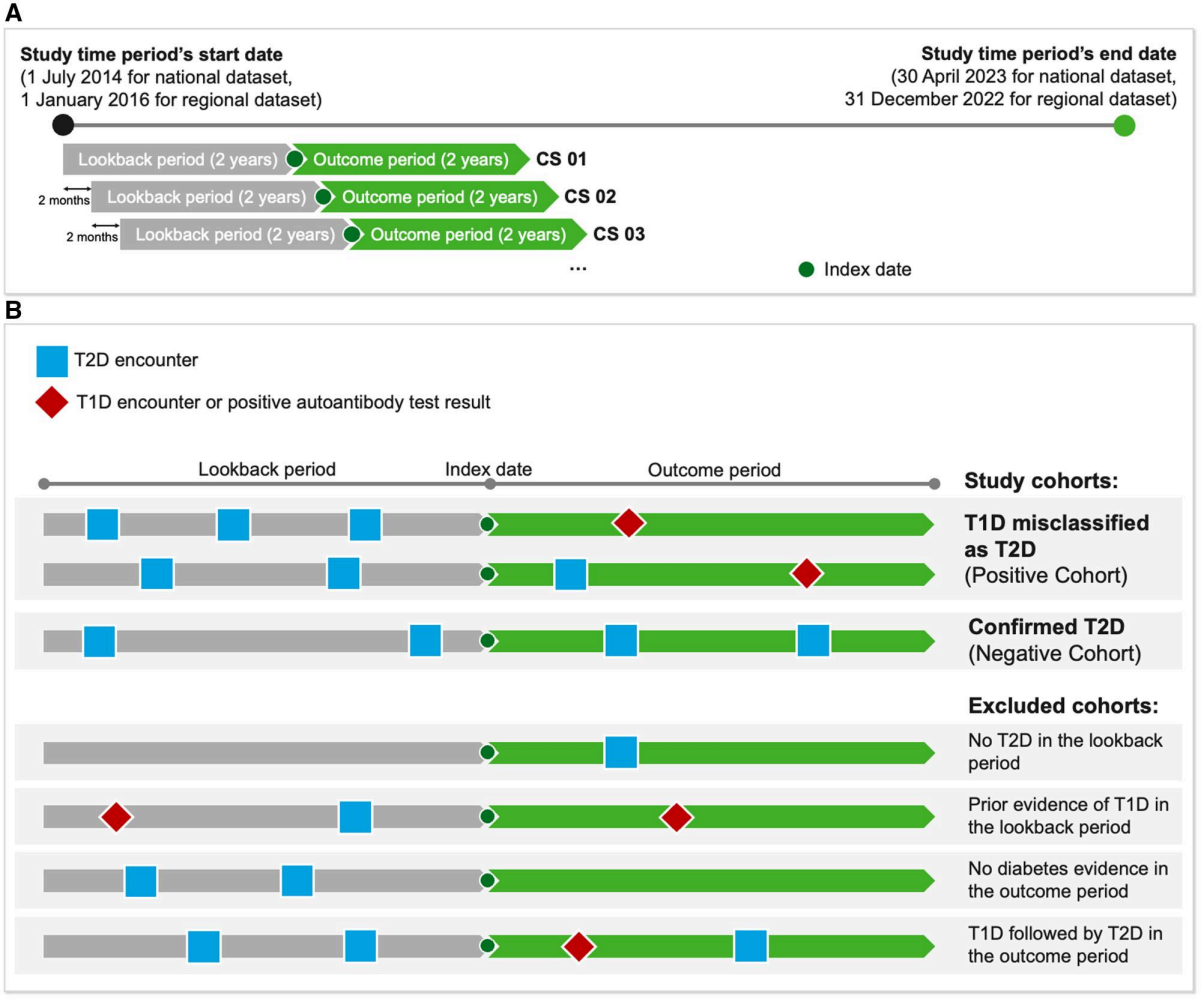
(A) Schematic representation of the rolling cross-sections generated across this study’s time periods. (B) Illustration of the positive and negative patient CSs defined for this study (top), as well as the cohorts that have been excluded (bottom), based on their sequence of T1D/T2D events in their lookback and outcome periods.

The model was first enhanced to score patients not only at their first T2D diagnosis (which would require real-time algorithm use at the point of care) but also at subsequent time points. Real-time use is challenging due to the time lags in linked HIE data, while the revisited algorithm can serve as a screening tool also outside the traditional office visits. This modification was achieved by dividing patients’ longitudinal data into time windows (*cross-sections* [CSs]), each made up of a lookback period, an index date, and an outcome period (see Figure 1A). Index dates were shifted by 2 months increments to generate multiple CSs per patient. This not only augments the training dataset but also reflects the model’s intended deployment scenario, where each diabetic patient would be periodically scored by the model using shifted representations of their evolving clinical journey. There was no requirement that a T2D encounter should be recorded at the index date, but at least one T2D encounter must be present in the lookback period for inclusion (see Figure 1B). Additionally, the outcome period length was reduced from 3 to 2 years. This allowed the algorithm to learn from more recent data and to increase sample size.

Finally, the cohort definitions were refined by, first, expanding the definition of T1D diagnosis to include positivity to T1D islet autoantibodies. Second, patient CSs with no evidence of either T1D or T2D during the outcome period were excluded, to avoid assumptions about their diabetes type when information was insufficient.

#### Feature engineering

Cheheltani et al^9^ implemented a feature engineering process that aggregated granular coding schemes and free text that refer to clinical predictors of interest. However, due to the discrepancies between the national and regional data, a more extensive aggregation exercise was necessary for this study to identify relevant predictors on both datasets. For instance, while medications are all coded using National Drug Codes (NDC) in the AEMR, the HIE dataset required mapping a combination of NDC, Generic Product Identifiers, RxNorm codes, and free-text fields to the same clinical events captured in the AEMR. The full list of predictors and their respective coding systems can be found in Table S1.

All predictors from the original model^9^ were retained for this work, with one exception: T2D diagnosing specialty features were excluded, since provider specialty information is typically absent in HIE datasets (including the HSX one).

#### Modeling

This phase involved 3 steps: (1) retraining the XGBoost model on national data using the adjusted cohorts and features, (2) externally validating this model on patient CSs from the regional dataset, and (3) training a localized model on regional data only. Both models were trained (steps 1 and 3) using binary cross-entropy as loss function. To compare the performance of the national model and the localized model on regional data (steps 2 and 3), 2 main changes were made to the original modeling approach.^9^

First, the recursive feature elimination strategy was omitted during training of both the national and the localized model, to ensure that they learned from the same set of predictors. However, features that showed minimal or zero predictive power (in step 1) were excluded from the study (see Table S1).

Second, for model localization (step 3), 5-fold nested cross-validation (CV) was adopted, randomly assigning each patient—with all their CSs—to a single fold. This allowed scoring all HIE patient CSs by both the national model (step 2) and 1 of 5 localized models (one for each outer CV loop, with hyperparameters optimized independently at each inner loop). CV predictions were aggregated to compute unified performance metrics and compare the localized model against the national one across the entire regional dataset. Before performing this aggregation, we confirmed consistent score distributions across folds (see Table S2), and a Cliff’s Delta analysis showed substantial overlap between scores of different validations sets (Table S3). We also verified that the 20 most predictive features (based on model gain) of the 5 trained models did largely overlap (Table S4).

### Evaluation metrics

Differences between the national and regional datasets were investigated by comparing patient cohort sizes and descriptive statistics for key predictors (focusing on their median value and non-missing proportion across patient CSs). The variability in data profiles received by different HIE members was also analyzed, by calculating the percentage of facilities sharing records for diagnoses, medications, vital signs, lab tests, and procedures.

The performance of both the national and localized models was assessed on the regional dataset, using the area under the receiver operating characteristic curve (AUROC), precision-recall (PR) curves, and the area under the PR curves (AUPRC). Special emphasis was placed on precision at low recall levels (5% and 10%), targeting a smaller population of highest scoring patients. This approach was driven by deployment considerations: clinical capacity for broad screening is often limited, hence the need to focus on a selected patient group with lower false-positive rate. 95% confidence intervals (CIs) for these metrics were estimated using bootstrap resampling (1000 iterations, resampling patients with all their CSs).

Additionally, a sensitivity analysis was performed to evaluate and compare the performance of the 2 models when scoring one single CS per patient (see the Supplementary Material).

Model interpretability was also investigated by analyzing feature importance, calculated as normalized total gain across the decision tree splits that the feature is used in. To examine their influence on predictions, features were binned and relative risk curves were generated by comparing average scores in each bin to a reference (baseline) bin.

## Results

In the national dataset, a total of 4 824 688 adult T2D patients were identified, of which 2 400 152 satisfied criteria to be included in the 2 study cohorts. From these patients, 113 933 positive (T1D initially classified as T2D) and 37 505 395 negative (confirmed T2D) patient CSs were generated (negative-to-positive ratio of 329:1). In the regional dataset, data from 243 946 adult T2D patients were used to generate positive and negative cohorts including 14 437 and 2 550 440 patient CSs, respectively (negative-to-positive ratio: 177:1).

**Figure 2.**
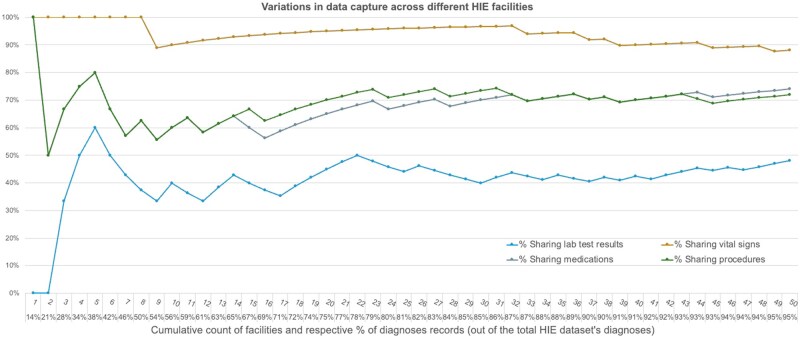
Facilities in the regional HIE are ranked by their number of diagnoses records in descending order. Each point along the *x* axis shows ranked facilities (up to the 50th facility) and the cumulative percentage of diagnoses that they cover (out of the total diagnoses records in the HIE dataset). The color-coded lines indicate the percentage of these facilities that also share lab test results (blue), vital signs (yellow), medications (gray), and procedures (green) records. For example, the first facility (ranking = 1) contains the highest proportion (14%) of diagnoses records out of the total HIE dataset; it shares also vital signs, medications, and procedures records; however, it does not share lab test results. The first 2 facilities (ranking = 2) account for 21% of the total HIE diagnoses; both facilities share vital signs records (100% on the *y* axis) and do not share lab test results (0%); however, only 1 of these 2 facilities (50%) shares procedures and medications.

In Table 1, key descriptive statistics for the study cohorts are presented. In both the national and regional datasets, the misclassified T1D patients were characterized by younger age, fewer T2D encounters, lower weight and BMI, as well as higher HbA1c values and prevalence of insulin usage, compared to the confirmed T2D patients. However, differences in the demographics of these 2 datasets could be observed. In the national dataset, Caucasian patients cover 84% and 81% of the positive and negative samples, respectively, with known race information. However, these percentages drop to 60% and 55%, respectively, in the regional dataset. Moreover, records of vitals (weight and BMI), lab test results (HbA1c), and prescribed medications (insulin) were found more frequently overall in the national dataset compared to the regional one. Out of 536 health facilities contributing to the HIE data pool, we found that only 415 share diagnoses records, which are crucial for identifying T2D patients and building cohorts for this study. Among these 415 facilities, 87% share medication records, 87% share vital signs, 68% lab test results, and 21% procedures. The amount of patient records covered by these facilities varies largely too. Notably, 50 large institutions alone contributed to 95% of all diagnoses in the regional HIE dataset (Figure 2): 74% of these 50 facilities share medication records, 88% share vital signs, 48% lab test results, and 72% procedures.

**Table 1.** Descriptive statistics for key demographic and clinical predictors used in this study.

		National dataset (AEMR)		Regional HIE dataset (HSX)	
		**Positive cohort** (*n* = 113 933)	**Negative cohort** (*n* = 37 505 395)	**Positive cohort** (*n* = 14 437)	**Negative cohort** (*n* = 2 550 440)
**Age** (median, in years)		60	67	60	66
**Gender** (% male)		46.5%	49.6%	50.4%	50.1%
**Ethnicity** (%)	Asian	1.3%	2.1%	2.5%	4.1%
	African American	8.5%	11.0%	26.2%	31.4%
	Caucasian	65.8%	65.6%	57.5%	53.1%
	Hispanic	0.3%	0.5%	1.8%	1.6%
	Other	2.0%	2.1%	7.7%	6.7%
	Unknown	22.1%	18.8%	4.3%	3.7%
**Count of T2D encounters** (median, in the lookback period)		3	5	2	3
**Weight** (median, in lbs [% of patient CSs with weight information])		192.8	205	182.6	196.6
		(97.5%)	(98.7%)	(65%)	(72%)
**BMI** (median, in kg/m^2^ [% of patient CSs with BMI information])		30.0	32.3	28.6	33.7
		(96.4%)	(98.1%)	(39.6%)	(45.5%)
**HbA1c** (median, in % [% of patient CSs with HbA1c information])		8.3%	7.2%	8.5%	7.5%
		(38.7%)	(46.7%)	(12.7%)	(10.5%)
**Insulin use** (% patient CSs with insulin prescriptions)		69.5%	25.4%	23.4%	11.2%

Descriptive statistics are stratified both by dataset (national vs regional HIE) and cohort (positive vs negative). Statistics for weight, BMI, and HbA1c are based on their most recent values with respect to index date, in the patient CSs where these measurements were available in their lookback.

Abbreviations: AEMR, ambulatory electronic medical records; BMI, body mass index; CSs, cross-sections; HIE: health information exchange; HSX, HealthShare Exchange; T2D, type 2 diabetes.

**Figure 3.**
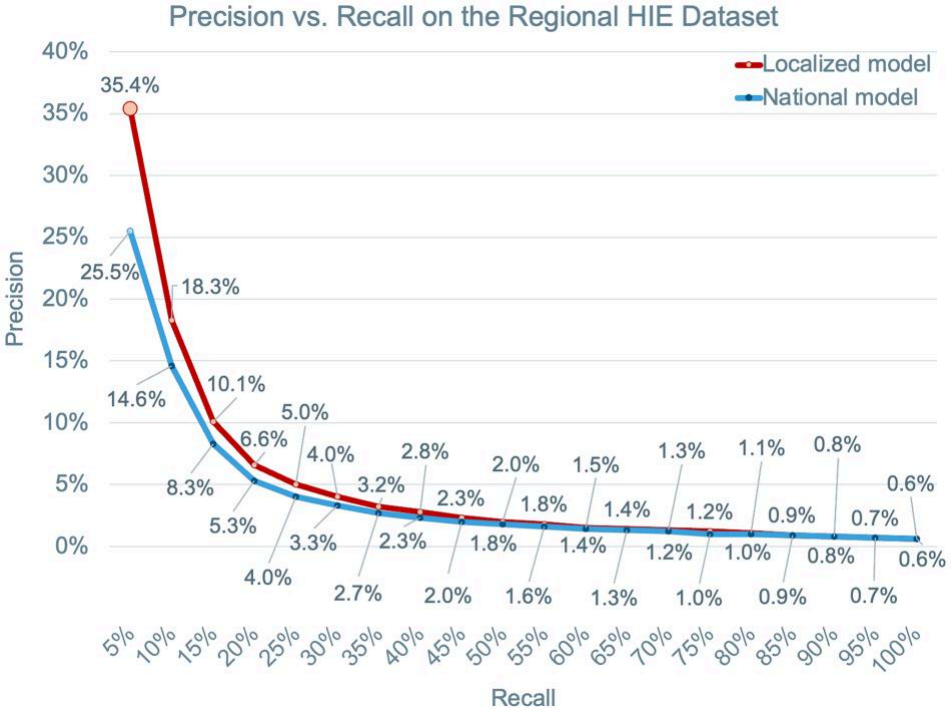
Precision-recall (PR) curves of the national (blue) versus localized (red) model applied on the regional HIE dataset. The PR curve for the localized model was obtained by combining all outer fold predictions after performing nested cross-validation.

**Figure 4.**
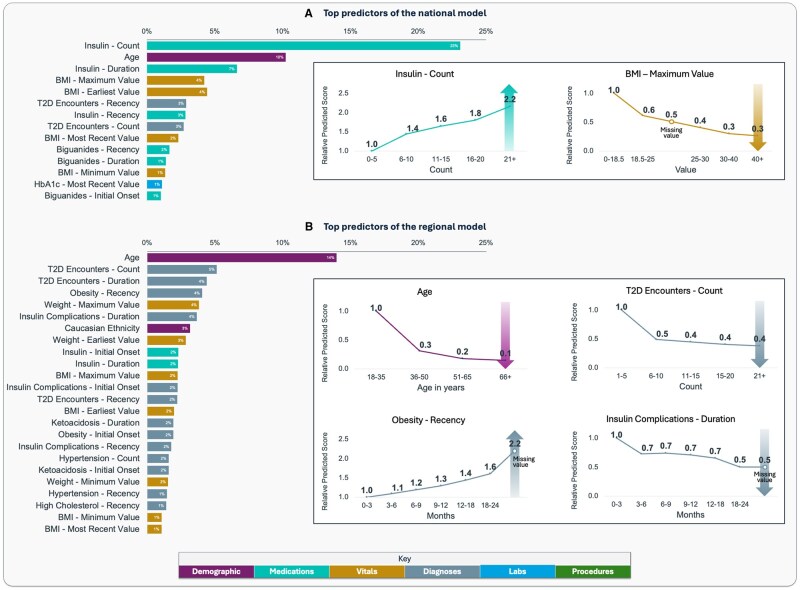
For both the national (A) and localized (B) model, all predictors with feature importance (normalized total gain) ≥ 1% are shown on the left-hand side and are color-coded based on the type of feature. For some selected predictors with high importance, their average impact on the output scores is estimated on the regional HIE dataset (white boxes on the right-hand side) for both the national (A) and localized (B) model using relative risk curves. Some features (BMI, recency of obesity diagnosis, and duration of insulin complications) can also have missing values (if that specific predictor cannot be found in the patient’s lookback period): in these cases, relative predicted risk scores are still represented in the graphs, even if they do not belong to any bin on the *x* axes.

The national model was re-trained on national AEMR data using optimized hyperparameters and a final set of 347 predictors. It was then validated on the regional dataset, characterized by an incidence of T1D reclassification of 0.56%. It achieved an AUROC of 0.751 (95% CI, 0.740-0.760), an AUPRC of 5.48% (ie, 10-fold improvement over baseline incidence; 95% CI, 4.64%-6.37%), and a precision of 25.5% (95% CI, 20.04%-32.70%) and 14.6% (95% CI, 11.8%-17.42%) at 5% at 10% recall, respectively (Figure 3). Therefore, if the algorithm was employed to screen the highest scoring patients—ie, covering 5% or 10% of the total reclassified population—an estimated 46-fold or 26-fold improvement in precision would be achieved compared to performing random screening on a portion of T2D population of equal size. As shown in Figure 4, features engineered from records of insulin prescriptions, patient’s age and BMI were identified in the top 5 most predictive features for this model.

Each patient in the HIE cohorts—together with their respective CSs—was randomly assigned to 1 out of 5 folds for nested CV. Given comparable T1D reclassification incidence (ranging from 0.56% to 0.57%) and consistent output distributions across all folds, an assumption was made that the 5 outer loop models were equivalent to each other (and to a single localized model that would be trained on the full regional dataset). Due to this assumption, all outer CV predictions were aggregated to compute unified model performance metrics. An AUROC of 0.774 (95% CI, 0.765-0.784) and AUPRC of 6.88% (12-fold improvement over incidence; 95% CI, 5.88%-7.97%) were estimated for this localized model, and Figure 3 shows that precisions of 35.4% (63-fold improvement over incidence; 95% CI, 27.7%-45.7%) and 18.3% (33-fold improvement; 95% CI, 14.4%-23.8%) were observed, respectively, at 5% and 10% recall. Patient age, together with features engineered from T2D encounters, obesity diagnoses and weight, were identified as the top 5 most predictive features (Figure 4).

## Discussion

### Implications of deploying the algorithm with and without localization

This study explores the feasibility of using an ML model to detect adult-onset T1D initially classified as T2D in HIE settings. To our knowledge, it constitutes the first study to test an ML-based solution for this task on real-world HCO datasets.

First, an existing algorithm^9^ trained on a national dataset was tested on a regional HIE dataset, requiring several adjustments for compatibility with the HIE dataset. These addressed both the lack of diagnosing specialty information and data latency constraints that could undermine adoption of an algorithm originally intended for use at the point of care. This highlights the importance of designing algorithms with downstream deployment considerations in mind,^24^ to ensure their successful uptake in clinical settings.

The revisited national model achieved satisfactory performance (measured by AUROC and PR curves), despite the differences between the national and regional datasets. This indicates the great potential of leveraging large-scale EMR data for developing clinical ML models. However, relying solely on a non-localized model assumes that the differences between the development and deployment population are negligible, when, in reality, disease prevalence, care patterns, and data quality vary across regions and HCOs. For example, this study’s regional dataset showed higher incidence of T1D reclassification and greater racial diversity compared to the national dataset. Moreover, considerable differences in data capture were identified among the health facilities that feed into the HIE data pool. While the model can adjust to these differences to some extent, its performance may still be constrained.

Model localization involved retraining on the regional dataset and resulted in improved AUROC (increased from 0.751 to 0.774), AUPRC (from 5.48% to 6.88%), and precision on the highest scoring patients (from 25.5% to 35.4% at 5% recall). Improved performance was observed with localization also when testing the models on only one CS per patient (Table S5). While certain features (eg, age and T2D encounter frequency) remained highly predictive in both the localized and national models, some differences were observed between the 2 models’ top predictors. The localized model’s most predictive features were largely derived from diagnosis records and weight/BMI measurements, whereas the national model placed higher importance on HbA1c results and prescriptions for insulin and biguanides. These findings align with the characteristics of the HIE dataset: patient cohorts are built using diagnostic data from member facilities that do not always provide comprehensive capture of other types of clinical events. Therefore, model retraining on HIE data may help compensate for these gaps in data capture, by capturing more signal from events that are more frequently shared across facilities.

The improved precision achieved by the localized model can reduce the false-positive burden, making the algorithm more appealing as a CDST for efficient patient screening. However, it is also crucial to acknowledge that localization can be effort-intensive and impact scalability. If every HIE (or individual HCO) required retraining of an ML model, the cost and time for widespread deployment could become prohibitive. Additionally, even if localization improved performance in this study, the overlapping 95% CIs for PR metrics show that the differences between models were modest, suggesting that in some scenarios the additional effort required for localization may outweigh its benefits. Models trained on large-scale national datasets may even outperform localized models in smaller regions with limited training data. Another potential concern is inequality in access to ML tools and to specialized ML engineers: localization efforts may inadvertently exacerbate inequalities in access to technologies that are reserved for well-funded organizations.

### HIEs as a path to scale: benefits and challenges

HIEs offer a valuable opportunity for widespread deployment of ML models for multiple reasons: data integration and diversity, centralized validation, and standardization through both interoperability (eg, HL7, FHIR) and terminology (eg, ICD-10, RxNorm) standards.^25^ Compared to individual HCOs, HIEs provide larger sample sizes for training and testing algorithms and better capture the clinical history of patients that attend multiple facilities within the same region. However, several challenges remain.

Despite standardization initiatives like the “Promoting Interoperability Program,”^26^ data variability still exists between HCOs due to differences in the implemented coding standards and in the types of data elements shared.^27^ This variability can impact algorithm performance and the quality of the patient cohorts flagged for screening. For instance, some patients may already have a positive autoantibody test on their medical chart, but this may not be visible in the HIE data or not coded in a standardized way. These limitations necessitate healthcare providers to manually verify evidence of T1D before conducting more in-depth chart reviews of high-risk patients. This could slow down the adoption of these tools in clinical settings, where provider capacity is already limited.

The shared nature of HIE data can also raise concerns about data ownership and governance. Establishing standardized data usage policies among multiple HCOs can be time-consuming. Furthermore, smaller or less mature HIE networks may lack resources for implementing AI systems or acting on flagged cases. Therefore, when planning HIE deployment, it is crucial to engage with member HCOs to customize model outputs according to their needs and operational constraints. This may involve, eg, setting model score thresholds (above which patients are flagged as high-risk) based on a trade-off between model precision and the HCO’s screening capacity.

### Limitations and future directions

As previously noted, a limitation of utilizing HIE datasets for validation lies in the data gaps across member HCOs, which can impact the quality of the predictors and the model’s outputs. Patients attending facilities that share more extensive data feeds will have more complete predictors to be fed as input to the prediction model. Moreover, while the localized model’s results were reported under the assumption that CV results would generalize to a single model trained on the full regional dataset, deviations in performance may arise when the model is trained on the full dataset and tested on recent data.

To address these limitations, prospective validation of the localized model is currently ongoing at 2 of HSX’s member HCOs, to assess its impact on health outcomes when used as screening tool. For this initial deployment, we apply the 10% recall threshold, as this provides a good balance between precision, recall and clinical capacity in the 2 participating HCOs. However, both quantitative (eg, model precision) and qualitative (eg, clinicians’ feedback) results will be collected to refine the threshold in the future, to optimize effectiveness while minimizing alert fatigue. The algorithm’s precision during deployment will be compared to that reported in this study to determine the generalizability of the findings. Moreover, we will investigate differences between the 2 HCOs in terms of model performance, high-risk patient profiles, and data capture.

Future research should incorporate cost-effectiveness analyses to quantify the financial and operational trade-offs of using ML models in clinical care, determining whether the benefits of improved precision and/or recall outweigh the associated costs. Furthermore, to support clinical workflow integration, user experience design will also need to be prioritized by providing actionable outputs to clinicians, supporting their decision-making, building trust in data quality, and integrating with existing tech standards (eg, SMART on FHIR) to minimize disruptions. To reduce alert fatigue, references and visualizations of the data elements driving the model results will need to be provided to clinicians, to support their decision-making without requiring significant chart review or cognitive effort. Finally, algorithmic fairness can be addressed by exploring whether patients flagged as high-risk by the algorithm and subsequently screened by clinicians exhibit any biases towards specific demographic subgroups, such as particular ethnicities or age groups.

## Conclusion

Our study highlights both the potential and the challenges of deploying ML models for high-risk patient identification at scale. While retraining on local datasets—more closely reflecting the deployment population—can improve screening precision, it also requires substantial resources and may affect scalability. Moreover, HIEs offer great opportunities for large-scale deployment, but data gaps must be carefully evaluated to avoid undermining providers’ trust in ML-based tools. Although this work focuses on detecting potentially misclassified adult-onset T1D, we believe that these learnings can also inform other applications, making them a valuable resource for future research in this field.

## Supplementary Material

ooaf133_Supplementary_Data

## Data Availability

The national AEMR and regional HIE data belong to IQVIA and HSX, respectively, and cannot be shared. Data processing and model training were run using IQVIA proprietary software packages. The trained model object can be made available upon request to the corresponding author.
