# Supplementary material for: Deploying machine learning models in clinical settings: a real-world feasibility analysis for a model identifying adult-onset type 1 diabetes initially classified as type 2
Source: JAMIA Open. 2025 Oct 26;8(5):ooaf133. doi: 10.1093/jamiaopen/ooaf133 (PMC12557313; doi:10.1093/jamiaopen/ooaf133)
Supplement: ooaf133_Supplementary_Data [file ooaf133_supplementary_data.docx]

# Study methodology details and additional results

A step-by-step description of the study methodology is presented, outlining details on both data preprocessing and training/testing of the proposed ML models for identifying type 1 diabetes (T1D) patients initially classified as type 2 diabetic (T2D). Additional results on intermediate analyses are also reported.

## Study cohorts

*Diabetic patient CS generation*

Both for the national ambulatory electronic medical record (AEMR) and the regional health information exchange (HIE) datasets, an initial cohort was generated by selecting all patients that, at any time during the respective dataset’s study period, were over 18 years old and had at least one in-person encounter for T2D.

The longitudinal data of these T2D patients was then split into time cross-sections (CSs), each made up of a 2-year lookback period followed by a 2-year outcome window (see Figure 1a in the main paper). Every CS was anchored to an index date, i.e., the last date of the lookback period. Multiple CSs were generated on a rolling basis, by shifting index dates by 2 months increments throughout the study period.

Stratification criteria were applied to all the identified patient CSs, by retaining only those that had:

1. At least one encounter for T2D during the lookback period; AND
2. No evidence of acromegaly, chronic pancreatitis, Cushing’s disease, cystic fibrosis, gestational diabetes, maturity-onset diabetes of the young, pancreatectomy procedures or pancrelipase medication use (since these conditions may present similar symptoms as T1D); AND
3. At least one encounter (for any activity) before the start of the lookback period; AND
4. Patient age at index date greater or equal to 18 years.

*Positive and negative cohorts*

The T2D and T1D-related activity in the generated patient CSs was analyzed, to identify the two cohorts of interest for this study (see Figure 1b in the main paper). The positive cohort, or “T1D misclassified as T2D” cohort, was made up of patient CSs with:

1. No encounters for T1D or positive autoantibody results within the lookback period; AND
2. One of the following criteria in the outcome period:
   1. At least one positive autoantibody result, OR
   2. At least one encounter for T1D, and the first of these T1D encounters is after the last T2D encounter (if any).

Conversely, the negative cohort, or “confirmed T2D” cohort, was made up of patient CSs with:

1. No encounters for T1D or positive autoantibody results within the lookback period; AND
2. No encounters for T1D or positive autoantibody results within the outcome period; AND
3. At least one encounter for T2D within the outcome period.

In these cohort definitions, T1D and T2D encounters refer to in-person visits associated with a T1D- or T2D-specific clinical code (ICD-9, ICD-10 or SNOMED), or visits that contain any type of diabetes-related clinical code and for which the diabetes type is revealed from the problem description’s free-text field.

The autoantibody lab test results that were used for these cohort definitions were those detecting autoantibodies to insulin, glutamic acid decarboxylase (GAD), islet antigen 2 (IA2) and/or zinc transporter 8 (Zinc-8).

*Conflicting cohort*

In addition to the positive and negative cohorts, a *conflicting cohort* was generated, where outcome periods present conflicting T1D and T2D diagnoses that do not clearly reveal whether the patient was reclassified as T1D. These are characterized by:

1. No encounters for T1D or positive autoantibody results within the lookback period; AND
2. At least one encounter for T1D, but no positive autoantibody results within the outcome period; AND
3. At least one encounter for T2D in the outcome period; AND
4. The last T1D encounter in the outcome period before the last T2D encounter.

In the national AEMR dataset, where provider specialty information is available, priority was given to the diagnoses received from endocrinology providers to re-assign, when possible, conflicting cohort CSs to either the positive or negative cohort: i.e., the above-described positive and negative cohort definitions were used but only using information recorded from endocrinology encounters.

## Feature engineering

Model features were engineered for each patient CS, based on the clinical events recorded during their lookback periods. Relevant clinical predictors (comprising diagnoses, medications, lab tests, procedures, family history, as well as weight and BMI measures) were first outlined by clinical experts (see Table S1). The clinical codes and/or text descriptions of each predictor were then listed to be able to identify these events from the EMR. Relevant diagnoses and family history were identified using a combination of ICD-9, ICD-10 and SNOMED codes; medications using National Drug Codes (NDC) in the AEMR, and a combination of NDC, Generic Product Identifiers (GPI), RxNorm codes and free-text descriptions in the HSX dataset; procedures using a combination of the Common Procedure Coding System (HCPCS) or Current Procedure Terminology (CPT); while the identification of lab tests, weight and BMI relied solely on matching text descriptions.

For each of the predictors (except for family history) identified in the CS lookback periods, four types of features were engineered: (1) *count* (how many times the predictor was recorded in the lookback); (2) *initial onset* (number of days between the index date and the first event for that predictor in the lookback); (3) *recency* (number of days between the index date and the last observed event for that predictor in the lookback); (4) *duration* (number of days between the first and the last observed event for that predictor in the lookback).

For lab test results, additional features were computed by extracting both their earliest and most recent value, the difference between these two values (*delta* feature), their rate of change (*rate* feature, i.e., delta divided by the number of days between the earliest and most recent measurement), as well as their minimum, maximum and average value across the lookback period.

For weight and BMI measurements, their minimum, maximum, earliest, most recent and delta features were computed, together also with the difference between their maximum and minimum recorded value in the lookback period.

One binary feature was then incorporated to represent family history of diabetes. Finally, the full feature set was completed by adding three types of demographic features: (1) gender, encoded as a binary feature; (2) age at index date; (3) racial category (i.e., White, African American, Asian, Hispanic, Other or Unknown), encoded with one hot encoding.

This process led to the creation of a total of 653 model features. The distribution of their values, together with their non-missing proportion across all patient CSs, was investigated and compared between positive and negative cohorts.

## Modeling

*National model*

The AEMR CSs were temporally split into a training set (comprising the first 16 CSs, indexed between 30 June 2016 and 31 December 2018) and a holdout set (the three most recent CSs, indexed between 31 December 2020 and 30 April 2021), as shown in Figure S1a. The model evaluation on a more recent holdout set aims to capture temporal variations in performance, which are to be expected in a real-world deployment scenario where a model is trained on historical data and tested on more recent, unseen data. Moreover, the two-year gap between the training and holdout sets’ outcome periods was ensured to avoid data leakage. Prior to modeling, negative patient CSs in the training set were also randomly down-sampled to a negative-to-positive ratio of 50:1 for the sake of computational efficiency.

An XGBoost binary classifier was then trained to identify T1D patients misclassified as T2D. Before training the model on the full training set, a temporal cross-validation (CV) strategy was employed to optimize its hyperparameters (learning rate, maximum tree depth, gamma regularization, minimum child weight, and number of estimators). Moreover, feature importance—computed as normalized gain—was analyzed for each predictor, and only those with importance ≥ 0.1% were preserved to train the model on the full training set. This process reduced the number of features from 653 to 347.

In the AEMR holdout set, the incidence of T1D reclassification was 0.22%, which is consistent with the incidence reported by Cheheltani et al. (0.23%) for the original model.^1^ The newly-trained model achieved a precision of 26.5% and 15.9% at 5% and 10% recall, respectively (see Figure S2), and an AUROC of 0.86. Both precision-recall curves and AUROC showed an overall improvement compared to the ones reported on the originally published cohorts and model (see Figure 2 of Cheheltani et al.^1^). We believe that this is due to two main reasons: (1) the more extended study time period, which provides more longitudinal data for the model to learn from; (2) the use of a rolling CS setup with CSs indexed also after the first T2D diagnosis, which augments the training samples further and gives the model the ability to learn from patients’ data at different stages of their clinical journey.

A final XGBoost classifier was then trained on all the available AEMR patient CSs (across the full study time period, now including also the above-defined holdout set) and tested directly on the external HIE cohorts without further testing on national AEMR data.

*Localized model*

Due to the shorter study time period available in HIE data, it was not possible to apply the same temporal splitting strategy used for the AEMR model. Therefore, each patient identifier—together with all their respective CSs—was assigned to one single group (out of five in total), and a nested 5-fold CV approach was used instead (see Figure S1b). In this way, all the generated patient CSs were scored once by one out of five XGBoost models (one for each outer CV loop). These models’ hyperparameters were optimized independently at each inner loop, while the feature set was kept the same as the one used for the national model to ensure that all models made use of the same predictors.

All outer CV predictions were aggregated to compute unified performance metrics for the localized model. To ensure validity of merging model scores from the five CV holdout sets, we first inspected their distributions by examining their means and multiple quantiles. As shown in Table S2, the distributions appeared consistent across holdout sets. Following this initial inspection, we performed the Kruskal-Wallis test to investigate whether there were statistically significant differences between the five model score distributions. This test was chosen due to the non-parametric nature of the data and for its suitability in comparing more than two groups. A p-value < 0.05 was obtained, suggesting statistical significance in the difference between the five sets of scores. However, due to the large sample size (>500k samples in each holdout set), we deemed relevant investigating the practical significance of these differences by focusing on the effect size. Cliff’s Delta was employed as an effect size measure, which quantifies how often a randomly selected value from one group is larger than a randomly selected value from a second group. This metric ranges from -1 to 1: values near 0 indicate large overlap between the two groups and, as a rule of thumb,^2,3^ an absolute Cliff’s delta < 0.15 is considered to indicate negligible effect size. As shown in Table S3, across all pairwise comparisons between holdout sets, the calculated absolute Cliff’s Deltas ranged between 0.009 and 0.131. These results indicate that the distributions of model scores from different holdout sets overlap substantially, thereby supporting the assumption that the models trained on different folds can be considered equivalent to each other.

This assumption was further validated by comparing the 20 most important features (based on model gain) of the five different holdout models. As shown in Table S4, a large overlap exists between the five lists of most predictive features, as well as the clinical events (predictors) used to engineer such features.

*Sensitivity analysis*

The use of a rolling CS methodology entails evaluating the model on multiple CSs per patients, which are temporally shifted and highly correlated. This approach closely reflects the intended deployment scenario, where diabetic patients are scored repeatedly using updated clinical data that evolves gradually over time. However, to ensure that the difference in performance between the national and localized model is not driven by these correlated samples, we conducted a sensitivity analysis whereby the model was tested on only one CS per patient. Specifically, one CS per patient was randomly selected across 1,000 iterations and key performance metrics (AUROC, area under the precision-recall curve [AUPRC], as well as precision at 5%, 10%, 15% and 20% recall) were computed at each iteration. The results were summarized in terms of mean and percentiles (5^th^, 50^th^, and 95^th^) across all iterations and presented in Table S5. AUROC values remained consistent with those reported in the main manuscript using rolling CSs, while an increase in both AUPRC and precision across all investigated recall levels was observed for both models. This improvement is attributable to the higher incidence rate (also reported in Table S5) in the sensitivity analysis, since confirmed T2D (negative) patients contribute more CSs over time. Importantly, despite the overall increase in performance metrics, the localized model consistently outperformed the national model in terms of precision, reaffirming the findings reported in the main manuscript.

*Model performance stratified by demographic group*

Due to demographic differences (age, gender and ethnicity) between the AEMR dataset (used to train the national model) and the regional HIE dataset, model performance was also stratified by demographic group. This involved recalculating AUROC and AUPRC for both the national and localized models, using only patient CSs from each demographic group individually. In addition, group-level precision was computed by applying a threshold corresponding to the full-dataset 10% recall level, which better reflects an operational threshold that is likely to be used during deployment for patient screening. As shown in Table S6, performance differences exist between demographic groups, but model localization improved all evaluated metrics except for AUROC and AUPRC in the Asian population, and AUPRC alone in the “Other” or “Unknown” ethnicity groups.

**Table S1.** List of all the predictors that have been analysed for this study. Every predictor (second column) is categorised by type (first column). The coding schemes (or free text fields in some instances) used to identify these events are reported in the third and fourth column for the AEMR and HIE datasets, respectively. The predictors that showed predictive power and were used to train the models presented in this study are marked with an asterisk (*).

| **Clinical event type** | **Predictor name** | **National AEMR coding system** | **Regional HIE coding system** |
| --- | --- | --- | --- |
| **Diagnosis** | T2D * | ICD-9, ICD-10 and SNOMED codes | ICD-9, ICD-10 and SNOMED codes |
|  | Abnormal weight loss * |  |  |
|  | Abnormal weight gain |  |  |
|  | Acute pancreatitis * |  |  |
|  | Alopecia areata |  |  |
|  | Autoimmune thyroiditis |  |  |
|  | Chron’s disease |  |  |
|  | Celiac disease |  |  |
|  | Chronic heart disease * |  |  |
|  | Chronic kidney disease * |  |  |
|  | Diabetes-related procedures codes |  |  |
|  | Diabetic retinopathy * |  |  |
|  | Disease of pancreas (unspecified) |  |  |
|  | Elevated blood glucose levels * |  |  |
|  | Family history of diabetes * |  |  |
|  | Heart failure * |  |  |
|  | High cholesterol * |  |  |
|  | HIV/AIDS |  |  |
|  | Hypertension * |  |  |
|  | Hypertriglyceridemia * |  |  |
|  | Insulin complications * |  |  |
|  | Juvenile arthritis |  |  |
|  | Ketoacidosis * |  |  |
|  | Kidney failure * |  |  |
|  | Metabolic syndrome |  |  |
|  | Multiple sclerosis * |  |  |
|  | Non-alcoholic liver disease * |  |  |
|  | Non-alcoholic steatohepatitis |  |  |
|  | Obesity * |  |  |
|  | Polycystic ovary syndrome * |  |  |
|  | Polydipsia * |  |  |
|  | Polyphagia * |  |  |
|  | Polyuria * |  |  |
|  | Primary adrenocortical insufficiency |  |  |
|  | Psoriasis * |  |  |
|  | Rheumatoid arthritis * |  |  |
|  | Retinopathy |  |  |
|  | Sleep apnoea (obstructive) * |  |  |
|  | Sleep apnoea (other) * |  |  |
|  | Stroke * |  |  |
|  | Thyrotoxicosis with diffuse goitre |  |  |
|  | Ulcerative colitis |  |  |
|  | Vision loss |  |  |
|  | Vitiligo |  |  |
|  | Weight reduction procedure codes * |  |  |
| **Medication** | Alpha glucosidase inhibitors | National Drug Codes | National Drug Codes, Generic Product Identifiers, RxNorm, free-text descriptions |
|  | Amylin analogues * |  |  |
|  | Antidiabetic combinations * |  |  |
|  | Antihyperlipidemic * |  |  |
|  | Antihypertensives * |  |  |
|  | Beta blockers * |  |  |
|  | Biguanides * |  |  |
|  | Bile acid sequestrants * |  |  |
|  | Calcium channel blockers * |  |  |
|  | Cardiovascular agents |  |  |
|  | Continuous glucose monitors |  |  |
|  | Dipeptidyl peptidase 4 inhibitors * |  |  |
|  | Diuretics * |  |  |
|  | Disease-modifying anti-rheumatic drugs * |  |  |
|  | Dopamine receptor agonists |  |  |
|  | Glucose elevating agents |  |  |
|  | Graves’ disease treatment |  |  |
|  | HIV/AIDS treatment |  |  |
|  | Hypothyroidism treatment * |  |  |
|  | Incretin mimetic agents * |  |  |
|  | Insulin pump * |  |  |
|  | Insulin * |  |  |
|  | Meglitinides * |  |  |
|  | Monoclonal antibodies |  |  |
|  | Nonsteroidal anti-inflammatory drugs * |  |  |
|  | Pancreatitis treatment |  |  |
|  | Salicylates |  |  |
|  | Sodium-glucose cotransporter inhibitors * |  |  |
|  | Sulfonylureas * |  |  |
|  | Thiazolidinediones * |  |  |
| **Procedures** | A1c measurement * | Current Procedure Terminology, Common Procedure Coding System | Current Procedure Terminology, Common Procedure Coding System |
|  | A1c home monitoring device * |  |  |
|  | Ambulatory continuous glucose monitoring |  |  |
|  | Artificial pancreas |  |  |
|  | Calibrator solution |  |  |
|  | Diabetes evaluation and management |  |  |
|  | Diabetes management programme |  |  |
|  | Diabetes self-management training |  |  |
|  | Endocrinology assay |  |  |
|  | Fasting blood glucose measurement * |  |  |
|  | Glucagon measurement |  |  |
|  | Glucagon tolerance test |  |  |
|  | Glucose monitoring device * |  |  |
|  | Glucose measurement (from blood) |  |  |
|  | Glucose measurement (not from blood) |  |  |
|  | Glucose tolerance test |  |  |
|  | Glucose measurement via fingerstick * |  |  |
|  | Insertion of implantable interstitial glucose sensor |  |  |
|  | Insulin home infusion |  |  |
|  | Insulin injection |  |  |
|  | Insulin measurement * |  |  |
|  | Insulin pump |  |  |
|  | Islet cell antibody test * |  |  |
|  | Ketone blood test |  |  |
|  | Lancets |  |  |
|  | Medicare diabetes prevention programme |  |  |
|  | Pancreatectomy |  |  |
|  | Pancreatic islet cell transplantation |  |  |
|  | Post-prandial glucose measurement |  |  |
|  | Proinsulin measurement |  |  |
|  | Removal of implantable interstitial glucose sensor |  |  |
|  | Renal function panel * |  |  |
|  | Urine test |  |  |
|  | Weight reduction procedure |  |  |
| **Lab tests** | A1c * | Free-text descriptions | Free-text descriptions |
|  | C-peptide * |  |  |
|  | Fasting glucose * |  |  |
|  | Glutamic acid decarboxylase antibodies * |  |  |
|  | Insulin antibodies * |  |  |
|  | Islet antigen 2 antibodies * |  |  |
|  | Zinc transporter 8 antibodies |  |  |
| **Vitals** | BMI * | Free-text descriptions | Free-text descriptions |
|  | Weight * |  |  |
| **Other** | Age (in years) at index date * | N/A (information available in demographic table) | N/A (information available in demographic table) |
|  | Gender * |  |  |
|  | African American race * |  |  |
|  | Asian race * |  |  |
|  | Caucasian race * |  |  |
|  | Hispanic race * |  |  |
|  | Other race * |  |  |
|  | Unknown race * |  |  |

*
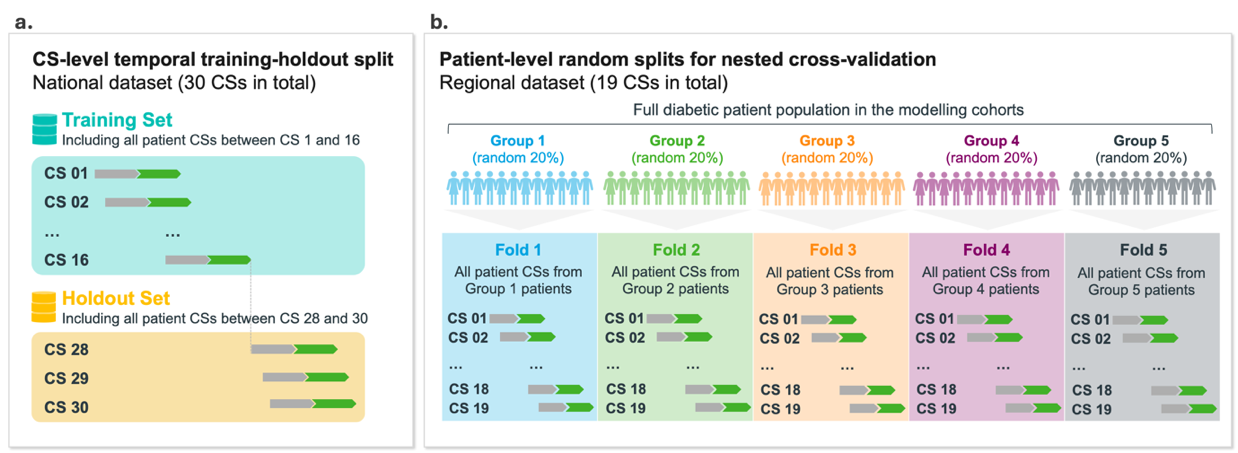
*

**Figure S1.** Schematic representation of the training-holdout data splitting strategy used for the development of the national model (a) and localized model (b).


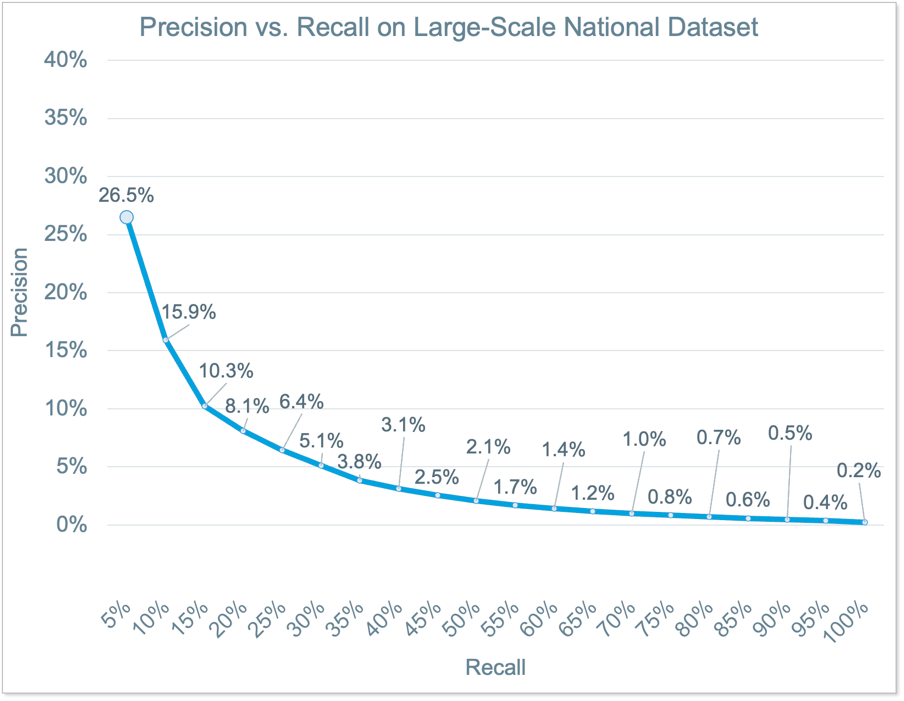


**Figure S2.** Precision-recall (PR) curves of the national model applied on the national holdout set.

**Table S2.** Distribution of model scores on all five outer loop models generated during model localization using nested cross-validation (CV).

|  | | **Model score** | | | | | | | |
| --- | --- | --- | --- | --- | --- | --- | --- | --- | --- |
| **CV holdout set** | **N Samples** | **Min** | **25%** | **50%** | **75%** | **95%** | **99%** | **Max** | **Mean** |
| **1** | 512,641 | 0.0002 | 0.0018 | 0.0031 | 0.0057 | 0.0141 | 0.0349 | 0.9418 | 0.0054 |
| **2** | 511,344 | 0.0003 | 0.0020 | 0.0034 | 0.0059 | 0.0148 | 0.0353 | 0.8901 | 0.0055 |
| **3** | 513,159 | 0.0006 | 0.0024 | 0.0037 | 0.0060 | 0.0145 | 0.0334 | 0.8951 | 0.0056 |
| **4** | 516,061 | 0.0002 | 0.0021 | 0.0034 | 0.0060 | 0.0149 | 0.0355 | 0.9137 | 0.0057 |
| **5** | 511,672 | 0.0002 | 0.0020 | 0.0033 | 0.0058 | 0.0147 | 0.0356 | 0.9806 | 0.0055 |

**Table S3.** Cliff’s Delta values obtained from the pairwise comparison of model scores across the five outer loop models generated during model localization using nested cross-validation (CV).

| **Pairwise Cliff’s Delta values** | | | | |
| --- | --- | --- | --- | --- |
| **CV holdout set** | **2** | **3** | **4** | **5** |
| **1** | -0.055 | -0.131 | -0.066 | -0.047 |
| **2** | - | -0.075 | -0.011 | 0.009 |
| **3** | - | - | 0.062 | 0.084 |
| **4** | - | - | - | 0.020 |

|  | **Engineered features with highest importance (top 20)** | | | | |
| --- | --- | --- | --- | --- | --- |
| **Clinical event** | **CV holdout set 1** | **CV holdout set 2** | **CV holdout set 3** | **CV holdout set 4** | **CV holdout set 5** |
| Age | Age at index date | Age at index date | Age at index date | Age at index date | Age at index date |
| T2D Encounters | Count  Duration  Recency | Count  Duration  Recency | Count  Duration  Recency | Count  Duration  Recency | Count  Duration  Recency |
| Weight | Max value  Min value  Earliest value  Most recent value  Max – min value | Max value  Min value  Earliest value | Max value  Earliest value | Max value  Min value  Earliest value | Max value  Min value  Earliest value |
| Insulin complications | Duration  Initial onset | Duration  Initial onset  Recency | Duration  Initial onset  Recency | Duration  Initial onset  Recency | Duration  Initial onset |
| Obesity | Count | Count  Recency | Count  Recency | Count  Recency | Recency |
| Ethnicity | Caucasian | Caucasian | Caucasian | Caucasian | Caucasian |
| BMI | Max value  Min value  Earliest value | Max value  Earliest value | Max value | Max value  Earliest value | Max value  Min value  Earliest value |
| Insulin prescriptions | Duration  Initial onset  Recency | Duration  Initial onset  Recency | Duration  Recency | Duration  Initial onset  Recency | Duration  Initial onset  Recency |
| Hypertension | Count |  | Count  Recency | Count | Count  Recency |
| Ketoacidosis |  | Initial onset | Duration  Initial onset | Duration | Recency |
| High cholesterol |  | Recency | Recency |  |  |

**Table S4.** For each of the five holdout sets from nested cross-validation (CV), the 20 features with highest importance (model gain) are listed (second to sixth column in the table). These features are grouped by the type of EMR clinical event (first column) from which they are engineered. A large overlap exists in the type of clinical events that were used to engineer the most predictive features across all five models.

**Table S5.** Results of the sensitivity analysis obtained by randomly sampling one CS per patient (from the regional HIE dataset) across 1,000 iterations. Incidence, area under the receiver operating characteristic curve (AUROC), area under the precision-recall curve (AUPRC), as well as precision at 5% (PR5), 10% (PR10), 15% (PR15) and 20% (PR20) recall were computed at each iteration. Their mean and 5^th^ (P5), 50^th^ (P50) and 95^th^ (P95) percentiles across all iterations are reported separately for the national (left) and localized (right) models.

|  | **National Model** | | | | **Localized Model** | | | |
| --- | --- | --- | --- | --- | --- | --- | --- | --- |
| **Metric** | **Mean** | **P5** | **P50** | **P95** | **Mean** | **P5** | **P50** | **P95** |
| Incidence | 1.11% | 1.10% | 1.11% | 1.12% | 1.11% | 1.10% | 1.11% | 1.12% |
| AUROC | 0.75 | 0.75 | 0.75 | 0.75 | 0.78 | 0.77 | 0.78 | 0.78 |
| AUPRC | 0.09 | 0.09 | 0.09 | 0.09 | 0.11 | 0.11 | 0.11 | 0.11 |
| PR5 | 38.5% | 35.7% | 38.3% | 41.6% | 54.5% | 50.0% | 54.4% | 59.4% |
| PR10 | 23.8% | 22.4% | 23.7% | 25.5% | 32.1% | 30.3% | 32.2% | 33.8% |
| PR15 | 16.1% | 14.9% | 16.3% | 17.0% | 20.7% | 19.4% | 20.8% | 21.8% |
| PR20 | 10.7% | 10.0% | 10.7% | 11.3% | 13.2% | 12.6% | 13.2% | 13.9% |

**Table S6.** Model performance stratified by gender, age group and ethnicity. For each subgroup of the population, its respective incidence (calculated as number of positive CSs over the total number of CSs), AUROC and AUPRC are calculated using CSs from that subgroup alone. On the right hand-side of the table, the subgroup precision obtained at the full-population 10% recall threshold is also reported. The metrics that were affected by a reduction after model localization are highlighted in bold.

|  | |  | **Subgroup AUROC** | | **Subgroup AUPRC** | | **Subgroup precision at full-population 10% recall threshold** | |
| --- | --- | --- | --- | --- | --- | --- | --- | --- |
|  |  | **Subgroup Incidence** | **National model** | **Localized model** | **National model** | **Localized model** | **National model** | **Localized model** |
| **Gender** | Female | 0.56% | 75.7% | 78.0% | 4.43% | 5.7% | 12.5% | 15.6% |
|  | Male | 0.57% | 74.5% | 76.8% | 6.75% | 8.2% | 17.1% | 21.3% |
| **Age group (in years)** | 18-29 | 5.02% | 83.0% | 87.1% | 30.5% | 39.2% | 21.4% | 23.1% |
|  | 30-44 | 1.43% | 80.0% | 83.0% | 11.4% | 14.9% | 14.0% | 17.9% |
|  | 45-59 | 0.62% | 73.7% | 76.6% | 3.7% | 4.1% | 10.9% | 16.0% |
|  | 60-74 | 0.45% | 71.3% | 73.5% | 1.8% | 2.0% | 9.2% | 10.0% |
|  | 75+ | 0.38% | 69.2% | 71.8% | 1.1% | 1.5% | 7.9% | 10.6% |
| **Ethnicity** | African American | 0.47% | 73.9% | 76.4% | 3.2% | 8.6% | 10.6% | 14.8% |
|  | Asian | 0.35% | 73.3% | **71.4%** | 1.7% | **1.6%** | 5.1% | 10.6% |
|  | Caucasian | 0.61% | 76.0% | 78.5% | 6.9% | 8.6% | 17.4% | 19.9% |
|  | Hispanic | 0.63% | 70.2% | 74.1% | 3.1% | 4.5% | 5.2% | 5.7% |
|  | Other | 0.71% | 71.2% | 74.6% | 7.1% | **6.5%** | 13.7% | 18.7% |
|  | Unknown | 0.66% | 73.8% | 76.1% | 5.3% | **4.5%** | 10.2% | 22.1% |

# References

1. Cheheltani R, King N, Lee S, et al. Predicting misdiagnosed adult-onset type 1 diabetes using machine learning. *Diabetes research and clinical practice* 2022; **191**: 110029.

2. Meissel K, Yao ES. Using Cliff’s delta as a non-parametric effect size measure: an accessible web app and R tutorial. *Practical Assessment, Research, and Evaluation* 2024; **29**(1).

3. Romano J, Kromrey JD, Coraggio J, Skowronek J. Appropriate statistics for ordinal level data: Should we really be using t-test and Cohen’sd for evaluating group differences on the NSSE and other surveys. annual meeting of the Florida Association of Institutional Research; 2006; 2006.
